# Antioxidative Effects of Black Currant and Cornelian Cherry Juices in Different Tissues of an Experimental Model of Metabolic Syndrome in Rats

**DOI:** 10.3390/antiox12061148

**Published:** 2023-05-24

**Authors:** Marija Paunovic, Jelena Kotur-Stevuljevic, Aleksandra Arsic, Maja Milosevic, Vanja Todorovic, Azra Guzonjic, Vesna Vucic, Snjezana Petrovic

**Affiliations:** 1 Group for Nutritional Biochemistry and Dietology, Centre of Research Excellence in Nutrition and Metabolism, Institute for Medical Research, National Institute of Republic of Serbia, University of Belgrade, 11000 Belgrade, Serbia; marija.paunovic@imi.bg.ac.rs (M.P.); aleksandra.arsic@imi.bg.ac.rs (A.A.); snjezana.petrovic@imi.bg.ac.rs (S.P.); 2 Department of Medical Biochemistry, Faculty of Pharmacy, University of Belgrade, 11351 Belgrade, Serbia; jelena.kotur@pharmacy.bg.ac.rs (J.K.-S.); azra.guzonjic@pharmacy.bg.ac.rs (A.G.); 3 Group for Neuroendocrinology, Institute for Medical Research, National Institute of Republic of Serbia, University of Belgrade, 11000 Belgrade, Serbia; mmilosevic@imi.bg.ac.rs; 4 Department of Bromatology, Faculty of Pharmacy, University of Belgrade, 11221 Belgrade, Serbia; vanja.todorovic@pharmacy.bg.ac.rs

**Keywords:** metabolic syndrome, polyphenols, black currant juice, cornelian cherry juice, oxidative stress, antioxidants

## Abstract

A Western-style diet, rich in fat and simple sugars, is the main risk factor for a significant number of chronic diseases and disorders, as well as for a progression of metabolic syndrome (MetS). One of the key mechanisms involved in MetS development is increased oxidative stress caused by the accumulation of body fat. Some dietary polyphenols have shown a protective role in preventing oxidative-stress-induced damage. We investigated the difference in the oxidative response of plasma, liver, and visceral adipose tissue in rats fed with a high-fat high-fructose (HFF) diet for ten weeks, and the effectiveness of polyphenol-rich juices (black currant (BC) and cornelian cherry (CC)) in HFF-diet-induced oxidative stress prevention. The most prominent impact of the HFF diet on redox parameters was recorded in the liver, whereas adipose tissue showed the most potent protection mechanisms against oxidative stress. Consumption of both juices decreased advanced oxidation protein product (AOPP) level in plasma, increased paraoxonase1 (PON1) activity in the liver, and significantly decreased total oxidative status (TOS) in adipose tissue. BC exerted stronger antioxidative potential than CC and decreased the superoxide anion radical (O_2_^•−^) level in the liver. It also reduced TOS, total antioxidative status (TAS), and malondialdehyde (MDA) concentration in adipose tissue. The multiple linear regression analysis has shown that the best predictors of MetS development, estimated through the increase in visceral adiposity, were superoxide dismutase (SOD), AOPP, TOS, and TAS. The consumption of polyphenol-rich juices may provide a convenient approach for the systemic reduction of oxidative stress parameters.

## 1. Introduction

The Western-type diet, characterized by food with a high percentage of fats and simple sugars, has resulted in a global pandemic of overweight and obesity. The development of metabolic syndrome (MetS), one of the main factors contributing to cardiovascular disease, is greatly influenced by obesity. One of the key mechanisms involved in this progression is the increased oxidative stress caused by the accumulation of body fat [1]. The accumulation of lipids in the liver and increased level of inflammation can cause the production of reactive oxygen species (ROS), leading to increased oxidative stress. The disturbance of cellular redox balance has harmful effects on cell homeostasis, structures, and functions and increases risk for the development of various pathologies [2]. To regulate the ROS levels, cells have an endogenous antioxidant defense system consisting of antioxidant enzymes and compounds with antioxidative roles [3,4]. Some nutrients can also contribute to compensatory antioxidant mechanisms, such as polyphenols [5]. There is substantial epidemiological evidence that foods and beverages containing phenol compounds have a strong potential to provide protective effects against various chronic diseases [6]. Black currant (*Ribes nigrum* L.) and cornelian cherry (*Cornus mas* L.) contain different biologically active phytochemicals, such as phenols, organic acids, and vitamins [7,8]. Since these components have been claimed to express antioxidative activity, consumption of black currant and cornelian cherry juices may be beneficial in preventing the redox imbalance. Recently published data demonstrated that black currant (BC) and cornelian cherry (CC) flavonoids positively affected several MetS risk factors, including dyslipidemia, hyperglycemia, and hypertension [9,10]. Diets with high fat and fructose promote the development of MetS in humans, as well as in rats, by deregulation of metabolic pathways in the adipose tissue and hypothalamus [11]. The aim of this study was to evaluate the effects of a high-fat high-fructose (HFF) diet on redox parameters in plasma, liver and adipose tissue, and potentially protective effects of polyphenol-rich juices.

## 2. Materials and Methods

### 2.1. Juice Sample Preparation

Commercial CC and BC juices, in three different series, were purchased from a local manufacturer. All juice samples were kept in a refrigerator at 4 °C before use. The samples were centrifuged at 14,000× *g* for 5 min at room temperature and filtered with a 0.45 μm filter (Macherey-Nagel GmbH & Co. KG, Düren, Germany) up to further analysis. Nutritional information, as well as the energy value of the juices, was obtained from juice labels (Table S1).

### 2.2. HPLC–MS/MS Determination of Phenolic Compounds in Juice Samples

HPLC–MS/MS analysis was performed according to the method described by Debetic et al. [12]. Briefly, Pursuit 3 PFP 150 × 4.6 mm, 3 μm column (Agilent Technologies, Amstelveen, The Netherlands) was utilized for chromatographic separations at a flow rate of 800 μL/min. A mobile phase consisting of solvent A (0.1% formic acid in water) and solvent B (acetonitrile) was employed with a gradient system elution: 0 min/5%, 22 min/35%, 24 min/100%, and 25 min/5%, followed by 5 min of column equilibration with 5% solvent B. The injection volume of the juices was 5 μL (both samples were diluted 10 times prior to the injection). Photodiode array detection was carried out in the UV range from 200 nm to 400 nm. After HPLC separation, eluent was introduced to the heated electrospray ionization (HESI) source of TSQ Quantum Access MAX triple quadrupole mass analyzer (Thermo Fisher Scientific Inc., San Jose, CA, USA). MS analysis was carried out in negative ionization mode along with MS parameters as follows: spray voltage, 2500 V; vaporizer temperature, 400 °C; capillary temperature, 300 °C; nitrogen; sheath gas (50 units) and auxiliary gas (10 units); helium; and collision gas at pressure of 1.5 mTorr. Peak integration and calibrations were replicated three times and performed using LC Quan™ software.

Individual phenolic compounds were identified by comparing their retention times and mass spectra with standards. Their quantification was performed with external calibration curves generated by integrating the area of absorption peaks.

### 2.3. Determination of Total Phenol, Flavan-3-ol and Anthocyanin Contents in Juice Samples

The total phenolic content was assessed using microassay as previously described [13]. Specifically, gallic acid (GEA) as the standard solutions and 50-fold-diluted juice samples were parallelly mixed with commercial Folin–Ciocalteu reagent and Na_2_CO_3_ and incubated for 60 min. Absorbance was measured at 630 nm (BIOTEK, Santa Clara, CA, USA, ELx800 Absorbance Microplate Reader) and expressed as mg gallic acid equivalents per liter of juice (mg GAE/L).

Determination of total flavan-3-ol content was performed using the p-dimethylaminocinnamaldehyde (DMAC) microassay [14]. Standard solutions of procyanidin B1 and 100-fold-diluted black currant apropos 10-fold-diluted cornelian cherry juices were mixed with DMAC solution. The reaction mixture in the plate was read against blank (ethanol) at 630 nm, and the results were expressed as mg procyanidin B1 equivalents per liter of juice (mg PB1E/L).

Total anthocyanin content was measured by the differential pH method [15]. Such anthocyanins determination takes into account their property to change color as a response to pH change. Thus, the absorbance of two dilutions for each juice sample (the first one in potassium chloride buffer (0.025 M, pH 1.0) and the second one in sodium acetate buffer (0.4 M, pH 4.5)) was read at 510 and 700 nm on a UV-vis J.P. SELECTA spectrophotometer (Barcelona, Spain). Total anthocyanin content was calculated based on formula: anthocyanin content (mg/L) = (A × MW × DF × 1000)/ε × L, whereby A = (A510 nm pH 1.0 − A700 nm pH 1.0) − (A510 nm pH 4.5 − A700 nm pH 4.5); MW = cyanidin 3-galactoside molecular weight (484.84); DF = dilution factor; ε = cyanidin 3-galactoside molar absorptivity in methanol/HCl (34,300 M^−1^·cm^−1^); L = cell path length (1 cm).

### 2.4. Animals and Treatment

The study was carried out in accordance with ARRIVE guidelines for laboratory animals, the National Statutes for Animal Rights (“Sl.gl.RS” 41/09 and 39/10), and the Directive 2010/63/EU. The study protocol was approved by the Ethics Committee of the Institute for Medical Research, National Institute of Republic of Serbia, University of Belgrade, Serbia, and Veterinary Directorate, Ministry of Agriculture and Environmental Protection, Republic of Serbia (No. 323-07-08846/2021-05), date 10 September 2021.

Thirty-six male rats, aged 3–3.5 months, were purchased from the Institute for Biological Research “Siniša Stanković”, Belgrade, Serbia. Following a 10-day acclimatization period, the animals were divided into four groups of nine rats each and placed on specific dietary regimens for a duration of 10 weeks. Each cage accommodated three rats.

The control group was maintained on a standard chow diet [16] and tap water, the HFF group was provided with a standard diet that was enriched with 25% sunflower oil, 20% fructose, and 0.1% cholic acid. The BC group was fed the HFF diet and provided with 20% cold-pressed black currant juice in tap water. The CC group was fed the HFF diet and provided with 20% cold-pressed cornelian cherry juice in tap water.

The animals were maintained in a controlled environment, characterized by a stable room temperature (21 ± 2 °C), humidity levels (55 ± 10%), and a light–dark cycle of 12–12 h. They were provided with unlimited access to food, water, and 20% juices in water. The animals’ weight, food, and liquid intake were recorded on a weekly basis.

### 2.5. Blood and Tissue Sample Preparation

The animals were anesthetized with 4% isoflurane, and blood samples were obtained via direct cardiac puncture with a 20 G needle. The samples were collected in EDTA (ethylene-diamine-tetra-acetic acid disodium salt) vacutainer tubes. Plasma was separated via centrifugation at 3000× *g* on 4 °C for 15 min, aliquoted, and stored at −80 °C for further analysis. Tissue samples of the liver and visceral adipose tissue were excised, rinsed with cold saline, and frozen at −80 °C for further analysis.

Liver and adipose tissue samples were homogenized in a glass tube on ice, using a manual homogenizer (Tissue grind pstl LC, Reynosa, Mexico) with 0.1 mol/L phosphate buffer at a weight-to-volume ratio of 1:9. The homogenates were centrifuged at 800× *g* for 10 min and 9500× *g* for 20 min at 4 °C to obtain the post-mitochondrial supernatant fractions, which were stored at −80 °C for further analysis.

The presence of MetS was confirmed through the blood analysis of lipid profile, glucose and insulin concentration, and blood pressure measurement.

### 2.6. Oxidative Stress Parameters

All analyses of oxidative stress parameters were performed using the ELISA reader (Pharmacia LKB, Wien, Austria) or ILab 300+ (Instrumentation Laboratory, Milan, Italy).

#### 2.6.1. PAB Determination

The pro-oxidative–antioxidative balance (PAB) was assessed through a modified PAB assay [17] that utilized 3,3′,5,5′-tetramethylbenzidine (TMB) as a chromogen. The assay is based on the reaction TMB and hydrogen peroxide and antioxidants. Standard solutions were created by combining different amounts (0–100%) of 1 mmol/L hydrogen peroxide with 6 mmol/L uric acid. The absorbance was measured at 450 nm. The values of PAB are expressed in arbitrary units and represent the quantity of hydrogen peroxide in the standard solution.

#### 2.6.2. SHG Determination

The levels of sulfhydryl (SH) groups were quantified using a method developed by Ellman [18]. This method involves the reaction of 2-nitrobenzoic acid with aliphatic thiols to form a yellow-colored p-nitrophenol. The levels of SH groups were determined spectrophotometrically by measuring the absorbance of the formed yellow reaction product at 412 nm. The results are expressed in units of mmol/L plasma/tissue homogenate.

#### 2.6.3. IMA Determination

Ischemia-modified albumin (IMA) was measured in plasma by the modified method of Bar-Or et al. [19]. The procedure of the assay involved the addition of 0.1% cobalt chloride to plasma or tissue prepared sample, followed by incubation to allow sufficient binding between cobalt and protein. After incubation, dithiothreitol (DTT) was added, and after two minutes the reaction was stopped with saline solution. The color development with DTT was evaluated using a SPECTROstar Nano UV/VIS spectrometer, and values are reported as absorption units (ABSU).

#### 2.6.4. MDA Determination

The levels of malondialdehyde (MDA) were quantified using a method developed by Girotti et al. [20]. The procedure involved mixing homogenized samples with 0.375% thiobarbituric acid (TBA), 15% trichloroacetic acid, and 0.25 mol/L HCl and incubating them at 100 °C for 5 min to allow MDA to react with TBA and form a red-colored complex. After cooling on ice, samples were centrifuged at 10,000× *g* for 10 min, and complex absorbance was measured spectrophotometrically at 535 nm. The concentration of MDA is expressed in units of µmol/L of plasma/tissue homogenate.

#### 2.6.5. PON1 Determination

The enzymatic activity of serum paraoxonase1 (PON1) was assessed through kinetic measurements using paraoxon and diazinon-O-analog as substrates following the Richter and Furlong method [17]. The speed of substrate conversion is monitored kinetically at 405 nm. The results are expressed in units of U/L plasma/tissue homogenate.

#### 2.6.6. SOD Determination

Determining the activity of this enzyme in plasma and tissue homogenates was conducted in accordance with the original method developed by Misra and Fridovich [21], with certain modifications. The method is based on superoxide dismutase (SOD) ability to inhibit spontaneous adrenaline auto-oxidation in an alkaline environment. Activity of this enzyme is determined by measuring absorption of product created by oxidation of adrenaline at 480 nm. The outcomes are stated in U/L of plasma or tissue homogenate.

#### 2.6.7. Superoxide Anion Radical Determination

For superoxide anion radical (O_2_^•−^), the modified Auclair and Voisin method was used [22]. The method is based on the reduction of yellow-stained nitroblue tetrazolium (NBT) to blue formazan. The rate of superoxide anion generation was determined by the rate of reduced NBT formation. Absorbance was measured at 550 nm. The measurements of superoxide anion radicals are reported in units of μmol NBT/min/L.

#### 2.6.8. TAS Determination

Total antioxidative status (TAS) was determined according to Erel’s method [23]. The assay utilized hydrogen peroxide in an acidic medium to oxidize reduced 2,2-azino-bis(3-ethylbenz-thiazoline-6-sulfonic acid) (ABTS), resulting in a discoloration of the reagent. The intensity of the discoloration was proportional to the concentration of antioxidants in the sample. The results were measured at 660 nm. The TAS concentration is indicated in units of µmol/L of plasma/tissue homogenate.

#### 2.6.9. TOS Determination

Total oxidative status (TOS) was determined with a method described by Erel [24]. The assay involved the oxidation of the ferrous ion-o-dianisidine complex to ferric ion by oxidants present in the sample, with the intensity of color produced being proportional to the total amount of oxidant molecules present. Absorbance was measured spectrophotometrically at 560 nm. Units of µmol/L were used to express the concentration of TOS in plasma/tissue homogenate.

### 2.7. Statistical Analysis

The statistical analysis was conducted using the SPSS 18.0 software (SPSS, Inc., Chicago, IL, USA). The Shapiro–Wilk test was used to check the distribution normality. Due to deviation from the normal distribution, data are presented as a median (interquartile range, i.e., 25th–75th percentile), and accordingly, non-parametric methods are used (Kruskal–Wallis and Mann–Whitney U tests as post hoc tests were used). The level of significance for all analyses was set to *p* ≤ 0.05. For polyphenols, concentration data are presented as mean ± standard deviation (SD). Spearman’s nonparametric correlation was used for bivariate correlation estimation. Multiple linear regression (MLR) analysis with backward selection was used to define the most significant model of predictors for visceral fat % at the end of the study.

## 3. Results

### 3.1. Polyphenols Concentration in Juice Samples

The applied HPLC–MS/MS method has been found to be suitable for juice sample analysis in a total time of 30 min. Chromatographic separations of phenolic compounds were achieved following the optimized method previously reported [12]. The TIC (total ion chromatograms) and EIC (extracted ion chromatograms) data are presented in the Supplementary Materials (Figure S1).

The obtained results represent the concentration of three different phenol classes and eleven individual phenolic compounds in BC and CC juices. The analysis revealed that black currant juice exhibited a higher concentration of almost all polyphenols (Table 1).

The total polyphenols (TPC) in BC juice averaged 1.39 g GAE/L, while in CC juice, it was around 1 g GAE/L (Table 1). The level of total flavan-3-ol (TF3C) was 1.04 vs. 0.19 mg B1E/L, and total anthocyanin (TAC) was 89 vs. 51 mg Cy3GE/L (BC vs. CC juice). Interestingly, measured malic acid, tartaric acid, and gallic acid concentrations were higher in CC, 0.78 vs. 0.47 g/L, 0.1 g/L vs. non-detectable value, and 0.009 vs. 0.001 g/L, respectively. Conversely, BC was assessed with double the protocatechuic acid content and even triple the amount of citric acid. When it comes to specific flavan-3-ols, such as epigallocatechin, catechin, epicatechin, procyanidin B1, and procyanidin B2, they were quantified in BC, while in CC, they were below the level of detection. Finally, flavonol rutin was measured in ten times the quantity in BC compared to CC juice.

### 3.2. Consumption of CC and BC Juices

**Figure 1:**
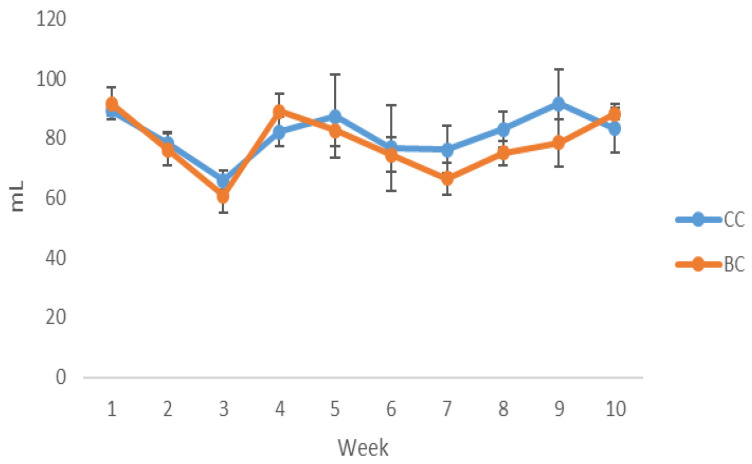
Daily intake of juices per cage during 10 weeks of treatment. CC—20% cornelian juice in tap water; BC—20% black currant juice in tap water.

The daily intake of CC and BC juices per cage during 10 weeks of treatment is shown in Figure 1. The intake of juices did not significantly change during the 10 weeks of treatment and was approximately 80 mL (20% juice in tap water) per cage daily for both groups.

### 3.3. Body Weight and Visceral Fat Percentage in Rats

**Figure 2:**
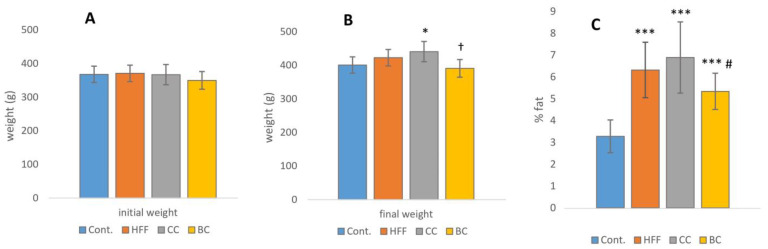
(**A**) Initial and (**B**) final weight in rats on different treatments. (**C**) Visceral fat percentage in comparison to total body weight in rats on different treatments; Cont.—control group (rats on standard chow diet and tap water); HFF—rats on diet enriched with 25% fat, 20% fructose, and 0.1% of cholic acid and tap water; CC—rats on HFF diet and 20% cornelian cherry juice in tap water; BC—rats on HFF diet and 20% black currant juice in tap water. * *p* ≤ 0.05, *** *p* ≤ 0.001 vs. control, # *p* ≤ 0.05 vs. HFF; † *p* ≤ 0.05 vs. CC at the end of the study.

The results of body weight and visceral fat percentage for the study groups are presented in the Figure 2.

The HFF diet did not induce changes in final body mass when compared with the control group. However, the percentage of visceral fat was significantly higher (*p* ≤ 0.001) in all groups consuming HFF (with or without juices) in comparison to the control. In addition, the rats consuming BC juices showed significantly lower visceral fat mass compared to those consuming only the HFF diet.

### 3.4. Oxidative Stress Parameters in the Plasma

The influence of polyphenol-rich juices on oxidative stress parameters in plasma is presented in Table 2.

The HFF diet significantly decreased PAB levels and did not affect other measured parameters compared to the control group. However, BC juice consumption reverted PAB to the values of the control group. Conversely, CC only slightly increased plasma PAB levels but significantly reduced the concentration of O_2_^•−^ and increased MDA levels compared to the HFF and BC group. Additionally, both juices decreased the plasma level of AOPP (measure of oxidative protein damage) relative to the control group.

### 3.5. Oxidative Stress Parameters in the Liver

The influence of CC and BC juices on oxidative stress parameters in rats’ livers is presented in Table 3.

The HFF diet significantly increased the activities of SOD and PON1, the level of TOS, and decreased the level of TAS in the liver compared to the control group. When BC juice was given with the HFF diet, it significantly increased TOS, PAB, AOPP, activity of PON1, and SOD, while decreasing O_2_^•-^ and TAS, compared to the control group. Compared to HFF alone, this juice also increased AOPP and PON1 and reduced the level of O_2_^•−^. On the other hand, CC juice given with the HFF diet increased PON1 and SOD levels and decreased MDA concentration in comparison to control. Relative to HFF, the CC group showed lower levels of MDA and higher levels of PON1 and TAS. When comparing the CC with BC groups, there were significantly lower TOS and AOPP, and significantly higher TAS levels in the CC group.

### 3.6. Oxidative Stress Parameters in Adipose Tissue

The influence of CC and BC juice consumption on oxidative stress parameters in visceral adipose tissue is presented in Table 4.

The HFF diet significantly increased the level of TAS in adipose tissue compared to the control group, while other parameters remained unchanged. However, when compared to the HFF group, the BC group exhibited decreased levels of TOS, MDA, PON, and TAS. Similarly, the CC group showed a decrease in TOS and IMA, as well as an increase in PAB and TAS/TOS range values, in comparison to the HFF group. Additionally, the CC group had lower concentrations of TOS and PON1 than the control group, which resulted in a higher TAS/TOS range. When comparing the two groups which consumed juices with the HFF diet, there was a significantly lower IMA concentration in the CC group.

### 3.7. Oxy Scores in Different Rats’ Tissues

**Figure 3:**
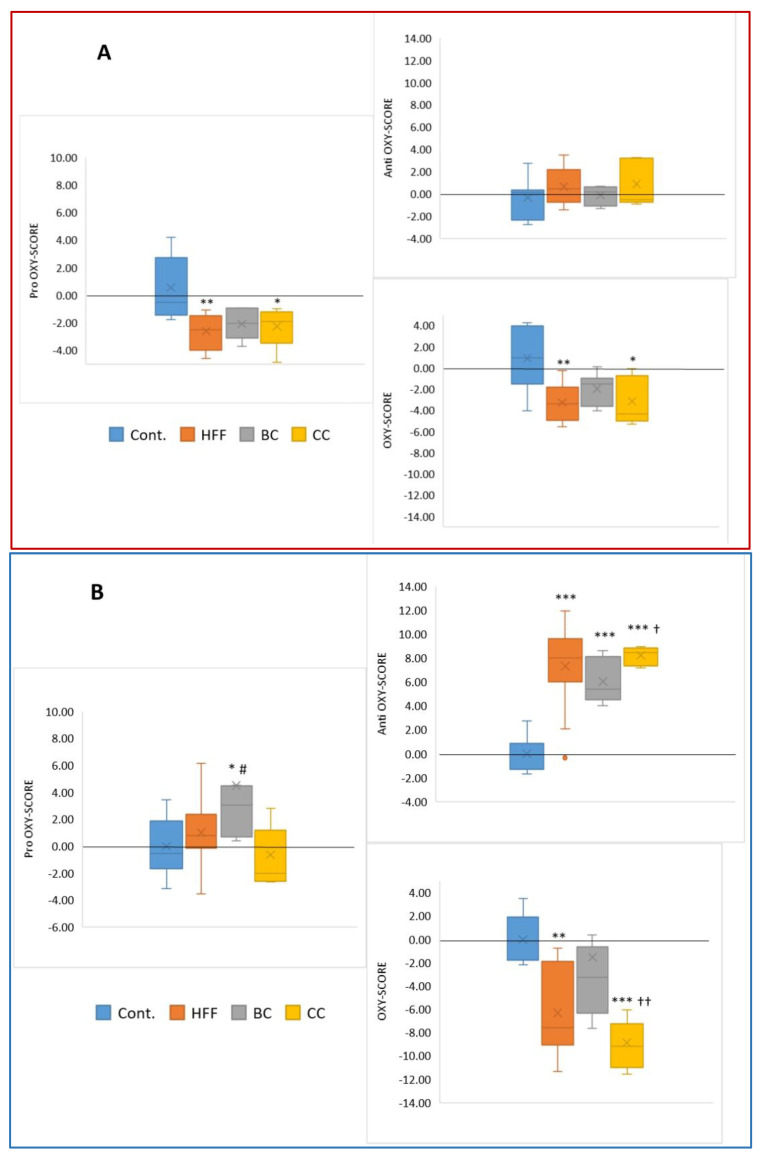

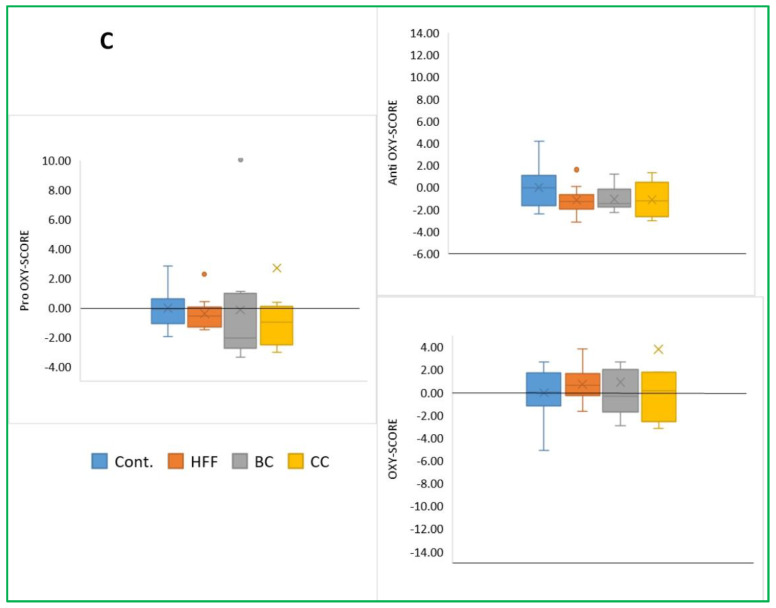
Oxy scores in different rats’ tissues (**A**) Pro-oxy, anti-oxy, and oxy scores in rats’ plasma. (**B**) Pro-oxy, anti-oxy, and oxy scores in rats’ livers. (**C**) Pro-oxy, anti-oxy, and oxy scores in rats’ adipose tissue; Cont.—control group on standard chow diet and tap water; HFF—group on high-fat high-fructose diet and tap water; BC—group on HFF diet and 20% black currant juice in tap water; CC—group on HFF diet and 20% cornelian cherry juice in tap water. * *p* ≤ 0.05 vs. control group, ** *p* ≤ 0.01, *** *p* ≤ 0.001 vs. control group; # *p* ≤ 0.05 vs. HFF group, † *p* ≤ 0.05, †† *p* ≤ 0.01 vs. BC group.

The oxy score represents the two primary components of oxidative stress: the build-up of oxidative damage and the decline in antioxidant defenses. Differences between pro-oxy, anti-oxy, and oxy scores in plasma, liver, and adipose tissue are presented in Figure 3.

The blood analysis showed the decreased levels of pro-oxidants (generated from fatty food) upon polyphenol-containing juice treatment. Blood antioxidants did not change under treatments; therefore, the oxy score remained similar to pro-oxy scores in all four experimental groups. The most intense redox score changes were in the liver, where BC treatment significantly increased the pro-oxy score, unlike CC treatment. Antioxidant content increased in all three treatment groups as a result of CC treatment. This was confirmed by a general fall in oxy scores, especially large in the CC group. Although there were several significant redox status parameter changes in adipose tissue, redox scores remained unchanged (i.e., balanced) in this tissue.

The plasma analysis showed significantly lower pro-oxy and oxy scores in the HFF and CC groups compared to the control group. There was no difference between the HFF group and the groups consuming juices. In the liver, a higher pro-oxy score was observed in the CC group compared to the control and HFF groups. Additionally, significant differences were found between the CC and BC groups. All three groups on the modified diet had higher anti-oxy scores than the control group, while the CC group had a higher anti-oxy score than the BC group. Finally, both the HFF and CC groups had a lower oxy score than the control group, and the CC group had a significantly lower oxy score than the BC group. There was no significant difference in pro-oxy, anti-oxy, or oxy scores in rats’ adipose tissue between groups.

### 3.8. Visceral Fat Percentage, Weight, and Oxidative Stress Parameters Correlation

There was a negative correlation between initial weight and weight change (∆weight) and a positive between final weight and ∆weight. The percentage of visceral fat positively correlated with ∆weight and final weight. Additionally, the plasma level of MDA positively correlated with initial and final weight (Table S2). The plasma level of PAB positively correlated with visceral fat and negatively with the SH groups. Both plasma levels of AOPP and IMA positively correlated with the SH groups’ concentration (Table S3).

The results of this study indicate a negative correlation between MDA level in the liver and final weight and PON1. The level of O_2_^•−^ negatively correlated with visceral fat, AOPP, and PON1. The activity of SOD had a positive correlation with visceral fat, TOS, and PON1 and a negative correlation with TAS/TOS. PON1 had a positive correlation with visceral fat (Table S4). Correlations between oxidative stress parameters are presented in Table S4.

A positive correlation between MDA and both TOS and TAS levels in adipose tissue was demonstrated, while a negative correlation was found between the level of AOPP and visceral fat. The level of IMA negatively correlated with final weight, PAB, and the TAS/TOS ratio and positively with TOS. Concentration of O_2_^•−^ positively correlated with AOPP and TOS and negatively with the TAS/TOS ratio. The SH group positively correlated with TOS and negatively with the TAS/TOS ratio (Table S5).

### 3.9. Multiple Linear Regression Analysis

MLR analysis (backward selection) was applied to identify what redox status parameters and kinds of treatment were the best predictors of visceral fat percentage at the end of the study.

The best model of plasma parameters predicted the final visceral percentage consisted of experimental group status (the most significant predictor in a model), SOD and PAB values explaining 56% of the fat ratio variability (Table 5). The best predictors of visceral fat % from the parameters measured in liver tissue were SOD, as the most significant predictor, and O_2_^•−^, with marginal significance in a model; this model explains 52% of fat content variability. The same method showed a more extensive model of predictors consisting of experimental group status, SOD, AOPP, TOS, and TAS levels. The model explained 57% of variability in visceral fat content (VFC), with the experimental group serving as the most significant predictor in a model (Table 5).

## 4. Discussion

This study investigated the differences in the oxidative response of plasma, liver, and visceral adipose tissue in rats fed with an HFF diet and the antioxidant potential of polyphenols from BC and CC juices in possible alleviation of the harmful effects of the HFF diet. The HFF diet, high in fat and fructose and mimicking the Western diet, induces overweight/obesity, hyperglycemia, hyperlipidemia, hyperinsulinemia, hepatic steatosis, increases inflammation, and oxidative stress [25,26] and promotes MetS.

Our results revealed that adipose tissue has the most potent protection mechanisms for oxidative stress control. Additionally, hepatic changes in oxidative stress parameters were more pronounced than those in other examined tissues. Namely, the HFF diet led to significantly increased activity of the PON1 only in the liver. Paraoxonase is an antioxidant enzyme closely associated with HDL and is widely distributed in many tissues, including the liver, brain, lung, heart, kidneys, small intestine, aorta, and others [27]. PON1 plays an important role in preventing microvascular complications due to oxidative stress and against various toxic molecules [28]. In MetS, plasma PON1 activity was found to be reduced because of decreased HDL concentration, and the severity of MetS is directly proportional to oxidative stress which inactivates PON1 function [29]. PON1 has anti-atherosclerotic, anti-inflammatory, and antioxidative properties that protect lipid membranes and LDL from oxidation and reduce the level of lipid peroxides [30]. PON1 activity could be regulated by several nutritional and pharmaceutical modulators. It also depends on genetic polymorphisms and redox balance [31]. A diet rich in trans-unsaturated fatty acids and with a high content of oxidized lipids reduces PON1 activity. On the other hand, unsaturated fatty acids may increase PON1 activity [32]. Additionally, the level of PON1 is closely related to the level of intracellular ROS, resulting in its increased activity as an antioxidative response to oxidative stress [33]. In line with this, the higher activity of PON1 noted in our study was probably induced by unsaturated fatty acids from sunflower oil in the HFF diet, as well as by the increased production of ROS caused by the diet, resulting in the maintenance of the MDA level (as the end product of lipid peroxidation in liver).

MDA, a marker of late phase of oxidative damage, is a toxic product of aldehydes from lipid peroxidation, and its high concentration is commonly found in MetS [34]. Lipid peroxidation leads to a decrease in cell membrane fluidity, and alters cellular functions, and leads to a further increase in oxidative stress.

The activity of SOD (one of the most powerful enzymatic antioxidants in cells) is usually downregulated in MetS. As a consequence, O_2_^•−^ accumulates in the mitochondrial intermembrane space and inhibits ATP production and reduces insulin secretion, leading to insulin resistance, the accumulation of visceral fat, and the development of MetS [35]. However, in our study, the HFF diet significantly increased SOD activity in the liver, which confirmed results from earlier studies that a high-fat high-fructose diet in rats increased intracellular ROS production and enhanced antioxidant defense in the liver [36]. Accordingly, the high activity of SOD induced by the HFF diet indicates a possible compensatory mechanism for a high ROS, especially O_2_^•−^ production, thereby maintaining the liver level of O_2_^•−^ unchanged.

In line with the external production of ROS induced by the HFF diet in different tissues [36,37], we found a high level of TOS, reflecting the overall higher pro-oxidants and lower TAS levels, the latter being parameter which represents several biomolecules with reducing antioxidative capacity (as all other antioxidants) in the liver of HFF group. As a consequence of altered oxidation status, the HFF diet led to a decrease in the overall antioxidative index (AOI), calculated as the TAS/TOS ratio in the liver. Our results are in line with results presented by Selvi et al., who found significant increase in TOS and reduced TAS in the liver induced by both high-fructose- and high-fat-fed rats. Contrary to the results of the current study, they observed increased MDA levels in the liver induced by an HFF diet [38].

An excessive generation of free radicals in the cells induces the activation of not only antioxidant enzymes but also endogenous enzymatic and non-enzymatic antioxidant systems, which protect the cells against oxidative stress [39]. In line with these, our results revealed an increased level of TAS in visceral adipose tissue as an induced compensatory mechanism for the neutralization of ROS.

We also determined oxy scores, which present the difference between all pro-oxidant and all antioxidant scores. According to our results, the HFF diet led to significant OS decrease in the liver compared with the control, despite an increase in TOS and a decrease in TAS. A possible reason could be due to the increased activities of other antioxidant enzymes in the liver (not determined in this study) as a way of protection from pro-oxidants produced by HFF and fat accumulation.

Even though changes in oxidative stress parameters induced by the HFF diet were small, we have examined PAB as a potentially better representative of the natural balance between pro-oxidants and antioxidants, which is established in each physiological system. In our study, the plasma level of PAB was decreased by the HFF, while this parameter stayed stable in the liver and adipose tissue. The PAB presents hydrogen peroxide and other similar pro-oxidants and uric acid along with the similar reducing substances [40]. A lower level of PAB is probably the result of a higher level of uric acid (product of fructose metabolism), confirmed also in another study [41]. Since there is evidence that uric acid can induce both cytosolic and mitochondrial oxidative stress [42] and is associated with cardiovascular events and diseases [43], its decrease in plasma would be beneficial. In the literature, uric acid was described as both pro-oxidant and antioxidant and can act as a potent scavenger of reactive oxygen species. In contrast, in some cells, uric acid can generate free radicals [44]. Therefore, although the reduction of PAB is generally beneficial, the HFF-induced PAB decrease in this study is a consequence of elevated uric acid and thus may be harmful in some conditions.

A positive correlation between SOD and PON1 revealed in this study indicates a positive relationship between the HFF diet and oxidative defense system in the liver, already described for other tissues [45]. In addition, a positive correlation between SOD and TOS showed that a high level of pro-oxidative parameters leads to elevated activity of antioxidative enzymes. A negative correlation between SOD and TAS/TOS ratio suggests that a high level of AOI reduces the need for increased activity of antioxidative enzymes.

We also observed a positive correlation of TAS level with MDA level in adipose tissue, indicating that the level of lipid peroxidation in adipocytes increased with total antioxidative status. This may be a consequence of a compensatory increase in reducers, whereby one compound is oxidized while another is simultaneously reduced, and the resulting change may be measured as TAS. Nevertheless, these findings are controversial and need to be confirmed in further research.

A negative correlation of plasma PAB and percentage of fat indicated a close link between pro-oxidative–antioxidative balance and overweight and obesity. This could be a consequence of rapid decomposition of H_2_O_2_ into more toxic hydroxyl radicals and reactive free radicals, reflected in a decrease in PAB activity. Simultaneously, in adipose tissue, the hydroxyl radicals were observed to oxidize lipids, although their concentration was not measured.

The findings from this study indicate that the HFF diet leads to oxidative stress burden in the liver. It was demonstrated by increased levels of TOS and decrease of TAS levels and oxidative stress index. However, the antioxidative enzymes such as SOD and PON1 were also higher in the liver in the HFF group providing at least some protective equilibrium. Regarding plasma and adipose tissue, the overall influence of the HFF diet on oxidative parameters was neglectable.

### BC and CC Effects

It is known that polyphenols from juices play a key role as a free radical scavenger and that it protects against lipid and protein damage induced by oxidative instability. Consuming BC and CC juices, in this study, resulted in reducing oxidative stress in different tissues in rats fed by the HFF diet.

Consuming juices together with the HFF diet leads to further PON1 activity increase in the liver. Similar to the findings of this study, Francik et al. reported a significant increase in PON1 activity in plasma and brain tissue after supplementation of cornelian cherry fruits in addition to a high-fat diet and high-fructose diet [46]. Several recent studies reported a significant increase in liver PON1 activity [47] or no effect on plasma PON1 activity in mice supplemented with pomegranate juice in combination with a high-fat diet [48]. On the contrary, in this study none of the juices affected plasma activity of PON1 and they even decreased PON1 activity in adipose tissue. These variations in studies suggest that the type of juices, duration of the studies, and animal models may affect findings. The increase in PON1 activity protects the liver from oxidation damage caused by oxidative stress.

Despite the increased SOD activity induced by HFF in the liver, BC and CC juices did not affect it in all examined tissue. However, these juices influenced the concentration of O_2_^•−^ in other tissues. Namely, while BC juice decreased the level of O_2_^•−^ in the liver compared with the HFF and control groups, CC juice decreased its level in the plasma compared with the HFF and BC groups. In biological systems, polyphenols from juices act in different ways. They can break the lipid peroxidation process, scavenge H_2_O_2_ and convert it to H_2_O, decrease superoxide anion production, and modulate the activities of enzymes including the antioxidative defense [49]. Decreases in the level of O_2_^•−^ in plasma and liver without changes in SOD1 activity in this study indicate the strong antioxidative potential of both juices and their ability to scavenge O_2_^•−^. Nevertheless, it is indicative that SOD was a part of all three MLR models, but as a predictor in a model showed statistical significance only for tissues. This should not be surprising because this is a cellular enzyme, and its activity is connected with cells rather than to extracellular compartments. SOD activities did not differ in animals with higher visceral adipose tissue content compared to those with lower content (taking 75 percentile VFC as a cut-off value). However, this activity was significantly higher in adipose tissue of animals with higher VFC compared to animals with lower VFC. This speaks in favor of increased antioxidants’ protection with exaggerated oxidative stress in adipose tissue compartments [50]. The most important MLR result explaining the relation between oxidative stress and metabolic syndrome development, estimated through the visceral adiposity uplifting, reflected the best predictors model for adipose tissue. This involved AOPP, TOS and TAS, besides SOD. All four redox status parameters remained significant in a model predicting VFC increase. The adipose tissue parameters’ change increased with increased VFC (for AOPP and TOS) and was unaltered for TAS, which implies a significantly lower TAS/TOS ratio in adipose tissue of animals with the highest VFC, higher than the 75th percentile for the whole group. This leads us to conclude that the oxidative processes with the highest activity are in adipose tissue especially that with visceral fat dominance, and that redox disbalance has a role in visceral fat build-up, probably forcing polyunsaturated bonds oxidation.

On the other hand, consuming both juices together with the HFF diet did not affect TAS and TOS liver levels. The opposite results were presented by Celep et al. who studied antioxidant activity of 80% methanolic extract of *Cornus mas* L. leaves in normal, healthy rats and found that the total antioxidant capacity of liver was increased, without changes in the activity of antioxidative enzymes and hepatic lipid peroxidation [51]. However, BC juice in combination with the HFF diet decreased the high level of TAS induced by the HFF diet alone in the adipose tissue. More importantly, both juices led to a significant decrease in the level of TOS in adipose tissue, suggesting the strong antioxidative potential of these juices and indicating the different responses of tissues to antioxidant supplementation.

Although the HFF diet did not influence the level of MDA in the examined tissues, juice consumption along with the HFF diet led to its changes. Notably, BC juice led to a significant decrease in MDA level in visceral adipose tissue and non-significant in the liver compared with HFF, while CC juice showed a decrease only in the liver compared with HFF and control. A decrease in TOS representing the levels of H_2_O_2_ and hydroperoxide and a decrease in the level of MDA (as the end product of lipid peroxidation) indicated the strong potential of BC and CC juices to scavenge or neutralize the LOO. These results are in accordance with previous studies showing that treatment of rats with CC fruit extract significantly ameliorated the alterations induced by carbon tetrachloride in lipid peroxidation in the kidney [52]. Furthermore, dietary intervention with BC significantly reduced the increased concentration of TBARS and lipid hydroperoxides in the testes of rats exposed to diesel exhaust emissions [53]. Additionally, there was a surprising increase in the plasma level of MDA induced by CC juice compared with the HFF group and BC group. The analyses of juices in our study showed a high content of total polyphenols and total flavan-3-ol content which possess ROS-scavenging activity against LOO and O_2_^•−^. These contents were higher in BC than in CC, indicating higher antioxidative potential of the former and suggesting the protective role of black currant juice on lipid peroxidation in adipose tissue and plasma.

Cellular targets of ROS and RNS in tissues include the highly concentrated proteins that undergo post-transcriptional oxidative modifications at specific amino acid residues. Some of these modifications are reversible, while others are irreversible and result in impaired protein expression and activity that affect cellular transport and redox signaling. AOPP, as a measure of highly oxidative protein damage in plasma, is the end product of a reaction between albumin and hypochlorous acid. The formation of AOPP can lead to tissue damage and is closely associated with endothelial dysfunction and the development of atherosclerosis. An increase in the level of AOPP has already been reported in subjects with MetS [54]. A favorable effect that was exerted by both juices resulted in a decrease of the AOPP level compared with the control group. Thus, both juices displayed strong antioxidative features and protective effects on plasma proteins. Similar observations for freeze-dried fruit CC were reported by Francik et al. in rats fed with a high-fructose diet [46]. Contrarily, BC juice given with the HFF diet increased the level of AOPP in the liver compared with all other groups, HFF, control, and CC. The reason why the BC juice showed different effects on plasma and liver is not clear, but it can be assumed that an AOPP level in the liver is affected by some additional exogenous and endogenous factors, which can cause liver injury and AOPP generation [55]. These findings need to be confirmed in further research. On the other hand, the level of total protein SH groups (another marker for evaluation of oxidation of protein) decreased in plasma and adipose tissue but these differences were not significant.

Albumin is also sensitive to a high level of ROS and oxidative stress. Its modified form, albumin-AOPP, is also a representative marker of oxidative stress intensity and the degree of oxidative damage to proteins [56]. Moreover, the N-terminal sequence of human serum albumin is very susceptible to biochemical modifications and degradation induced by hydroxyl radicals, which lead to IMA formation. IMA increases in different diseases linked with ischemia and oxidative stress [57]. However, in our study the HFF diet did not induce changes in the level of IMA. BC juice given with HFF also did not affect IMA, but CC juice decreased the level of IMA in adipose tissue. There is evidence that gallic acid, which is found in CC juice (at a ten-fold higher concentration than in BC juice), is among the best peroxyl radical scavengers identified in lipid media [58], and it could explain why CC caused IMA reduction in adipose tissue.

Since adipose tissue is a very active endocrine organ, it is important to mention the role of adipokines such as leptin, visfatin, and adiponectin, which serve as markers for MetS, in oxidative stress pathology. The hyperleptinemia that occurs in MetS induces the production of ROS such as H_2_O_2_ and OH radicals, decreases the activity of cellular PON-1, and leads to increased plasma levels of MDA and hydroperoxides [59]. Similarly, visfatin, also elevated in MetS, has a pro-oxidant effect, inducing the production of O_2_^•−^ and increasing the activity of SOD and catalase [60]. Adiponectin, on the other hand, increases the production of NO and inhibits the release of ROS, but its concentration is lowered in MetS [61]. Besides reducing oxidative stress, supplementation with polyphenols restores altered adipokine expression, increases leptin sensitivity [62], decreases visfatin secretion [60], and increases plasma adiponectin concentration [63]. Therefore, supplementation with BC and CC juice could favorably affect MetS by changing adipokine levels.

It is also worth mentioning the role of gut microbiota in the prevention and treatment of MetS. A high-fat diet can contribute to MetS development by altering gut microbiota, while polyphenol intake can modulate its composition towards a healthy profile, having a beneficial impact against the metabolic disturbances in MetS [64]. In line with this, dietary patterns rich in fibers, such as a Mediterranean or a plant-based diet, in combination with polyphenol-rich juices, could provide a strategy to prevent and counteract MetS in humans [65]. Future studies are needed to confirm this hypothesis in MetS and its associated pathologies.

## 5. Conclusions

In conclusion, in MetS rat models induced by HFF, the pro-oxidative and antioxidative status of plasma, liver, and adipose tissues showed certain variations, with the liver affected the most. BC and CC juice consumption simultaneously with the HFF diet had a beneficial effect on redox status in different tissues in animal models of MetS. Generally, BC exerted stronger antioxidative potential than CC, although the CC-consuming group has shown lower O_2_^•−^ in plasma and higher AOI in adipose tissue. MLR revealed the best models for the visceral fat percentage prediction, for any of the three biological materials (blood, liver, and adipose tissue) that were used for analysis. High percentages of selected leading models influence (above 50%) speak in favor of significant oxidative stress and subsequent polyphenol juice treatment influence. Further studies on the effects of BC and CC juices in humans with MetS are warranted.

## Figures and Tables

**Table 1:** Phenolic compounds quantified in black currant and cornelian cherry juices.

**Total Contents**	**BC**	**CC**
TPC (mg GAE/L)	1392 ± 215	996 ± 95
TF3C (mg B1E/L)	1043 ± 60	187 ± 18
TAC (mg Cy3GE/L)	89 ± 10	51 ± 8
**Individual compounds**	**BC**	**CC**
Tartaric acid (mg/L)	ND	101.2 ± 8.7
Malic acid (mg/L)	472.2 ± 28.2	778.4 ± 47.5
Citric acid (mg/L)	1481 ± 169	50.8 ± 8.1
Gallic acid (mg/L)	1.1 ± 0.2	8.9 ± 1.9
Protocatechuic acid (mg/L)	0.3 ± 0.1	0.6 ± 0.1
Epigallocatechin (mg/L)	7.9 ± 0.1	ND
Catechin (mg/L)	6.0 ± 0.3	ND
Epicatechin (mg/L)	5.6 ± 0.3	ND
Procyanidin B1 (mg/L)	0.6 ± 0.1	ND
Procyanidin B2 (mg/L)	3.1 ± 0.4	ND
Rutin (mg/L)	0.30 ± 0.02	0.03 ± 0.01

BC—100% black currant juice; CC—100% cornelian cherry juice; TPC—total polyphenol content; GAE—gallic acid equivalent; TF3C—total flavan-3-ol content; B1E—procyanidin B1 equivalent; TAC—total anthocyanin content; Cy3GE—cyanidin 3-galactoside equivalent; ND—not detectable.

**Table 2:** Difference in redox status parameters caused by HFF diet, BC, and CC juice supplementation in rats’ plasma.

Parameter	Control Group	HFF	BC	CC	*p*
O_2_^•−^ (μmol NBT/min/L)	48 (37–51)	51 (44–57)	49 (46–55)	41 (39–47) #†	0.111
PAB (U/L)	118 (112–123)	96 (93–109) *	119 (112–126) #	109 (99–115)	0.029
MDA (µmol/L)	2.93 (2.59–3.19)	2.74 (2.52–2.89)	2.70 (2.44–3.04)	3.41 (2.89–3.70) #†	0.058
AOPP (µmol/L)	57.7 (53.6–61.2)	54.4 (45.9–63.1)	50.2 (46.4–53.9) **	49.9 (43.4–54.3) *	0.052
IMA (ABSU)	0.050 (0.046–0.060)	0.052 (0.046–0.057)	0.048 (0.045–0.057)	0.081 (0.039–0.301)	0.664
SOD (U/L)	141 (139–144)	141 (140–142)	141 (139–142)	142 (141–144)	0.639
SHG (mmol/L)	0.216 (0.173–0.222)	0.216 (0.190–0.262)	0.199 (0.182–0.225)	0.184 (0.148–0.277)	0.803

Data are presented as median (interquartile range) and compared with the Mann–Whitney U-test and Kruskal–Wallis test. HFF—group on high-fat high-fructose diet; BC—group on HFF diet and 20% black currant juice supplementation; CC—group on HFF diet and 20% cornelian cherry juice supplementation. * *p* ≤ 0.05 vs. control; ** *p* ≤ 0.01 vs. control; # *p* ≤ 0.05 vs. HFF; † *p* ≤ 0.05 vs. BC; NBT—nitroblue-tetrazolium; PAB—pro-oxidant antioxidant balance; MDA—malondialdehyde; AOPP—advanced oxidation protein products; IMA—ischemia modified albumin; SOD—superoxide dismutase; SHG—sulfhydryl groups; ABSU—absorbance units; *p*—*p* values obtained in the Kruskal–Wallis test.

**Table 3:** Difference in redox status parameters caused by HFF diet, black currant, and cornelian cherry juice supplementation in rats’ livers.

Parameter	Control Group	HFF	BC	CC	*p*
TOS (μmoL/L)	772 (686–815)	841 (794–890) *	887 (863–1228) **	777 (750–854) †	0.014
O_2_^•−^ (μmol NBT/min/L)	2440 (2310–2580)	2425 (2310–2760)	2230 (2000–2310) *##	2230 (2110–2440)	0.042
PAB (U/L)	226 (206–246)	234 (230–259)	259 (244–265) *	230 (209–237) †	0.060
MDA (µmol/L)	93.3 (83.0–119)	97.4 (88.2–120.7)	79.3 (60.7–103.7)	64.1 (54.1–81.8) *#	0.065
AOPP (µmol/L)	1381 (1355–1475)	1506 (1384–1604)	1741 (1676–1869) **##	1526 (1418–1680) †	0.003
IMA (ABSU)	0.543 (539–595)	0.622 (0.559–0.638)	0.654 (0.551–0.681)	0.591 (0.542–0.628)	0.234
PON1 (U/L)	0 (0–0)	85 (30–330) **	430 (360–490) ***##	400 (340–410) ***#	<0.001
TAS (μmoL/L)	14,010 (13,780–14,220)	11,345 (10,790–11,520) ***	11,420 (11,230–12,240) ***	13,160 (12,420–14,010) ###†	<0.001
SOD (U/L)	210 (0–240)	1260 (1180–1320) ***	860 (810–1290) ***	1320 (1300–1345) ***	<0.001
SHG (mmol/L)	8.71 (8.38–9.89)	8.60 (7.50–10.3)	8.44 (8.09–9.06)	8.27 (7.77–8.64)	0.589
TAS/TOS	18.4 (16.2–20.4)	13.2 (12.3–14.3) ***	12.6 (9.3–13.6) **	17.9 (15.3–18.2) ##††	<0.001

Data are presented as median (interquartile range) and compared with the Mann–Whitney U-test and Kruskal–Wallis test. HFF—group on high-fat high-fructose diet; BC—group on HFF diet and 20% black currant juice supplementation; CC—group on HFF diet and 20% cornelian cherry juice supplementation. * *p* ≤ 0.05 vs. control; ** *p* ≤ 0.01 vs. control; *** *p* ≤ 0.001 vs. control; # *p* ≤ 0.05; ## *p* ≤ 0.01; ### *p* ≤ 0.001 vs. HFF; † *p* ≤ 0.05; †† *p* ≤ 0.01 vs. BC; ABSU—absorbance units; TOS—total oxidant status; NBT—nitroblue-tetrazolium; PAB—pro-oxidant antioxidant balance; MDA—malondialdehyde; AOPP—advanced oxidation protein products; IMA—ischemia modified albumin; TAS—total antioxidant status; SOD—superoxide dismutase; PON1—paraoxonase1; SHG—sulfhydryl groups.

**Table 4:** Difference in redox status parameters caused by HFF diet, BC, and CC juice supplementation in rats’ visceral adipose tissue.

Parameter	Control Group	HFF	BC	CC	*p*
TOS (μmoL/L)	24.5 (22.0–27.5)	38.0 (24.0–39.0)	20.0 (14.5–20.0) ##	16.0 (14.0–19.0) *###	0.003
O_2_^•−^ (μmol NBT/min/L)	160 (150–160)	160 (150–160)	160 (145–160)	160 (150–160)	0.954
PAB (U/L)	386 (373–392)	380 (378–386)	385 (381–397)	388 (387–389) #	0.213
MDA (µmol/L)	11.5 (8.89–15.6)	13.7 (11.1–17.0)	9.26 (8.52–10.4) #	11.1 (7.04–12.6)	0.110
AOPP (µmoL/L)	376 (347–392)	319 (296–363)	298 (283–343)	319 (305–354)	0.169
IMA (ABSU)	0.528 (0.497–0.538)	0.526 (0.513–0.550)	0.542 (0.504–0.545)	0.486 (0.460–0.516) #†	0.103
PON1 (U/L)	15 (0–30)	10 (10–20)	5 (0–10) #	0 (0–0) *##	0.010
TAS (μmoL/L)	1955 (1830–2030)	2375 (2170–2450) *	2025 (1790–2245) #	2180 (2110–2180)	0.027
SOD (U/L)	1275 (1250–1310)	1225 (1210–1240)	1245 (1235–1275)	1240 (1200–1290)	0.221
SHG (mmoL/L)	0.319 (0.174–0.450)	0.334 (0.232–0.406)	0.246 (0.210–0.276)	0.218 (0.174–0.304)	0.254
TAS/TOS	75.4 (72.4–91.8)	58.6 (50.8–102.1)	107 (85.4–135)	132 (120–141) *##	0.010

Data are presented as median (interquartile range) and compared with the Mann–Whitney U-test and Kruskal–Wallis test. HFF—group on high-fat high-fructose diet; BC—group on HFF diet and 20% black currant juice supplementation; CC—group on HFF diet and 20% cornelian cherry juice supplementation; * *p* ≤ 0.05 vs. control; #, ##, ### *p* ≤ 0.05, 0.01, 0.001, respectively vs. HFF; † *p* ≤ 0.05 vs. BC. TOS—total oxidant status; NBT—nitroblue-tetrazolium; PAB—pro-oxidant antioxidant balance; MDA—malondialdehyde; AOPP—advanced oxidation protein products; IMA—ischemia modified albumin; TAS—total antioxidant status; SOD—superoxide dismutase; PON1—paraoxonase1; SHG—sulfhydryl groups; ABSU—absorbance units.

**Table 5:** MLR analysis for the association of visceral fat % and oxidative stress parameters in rats’ tissues.

**Plasma**				
Predictors	Dependent variable visceral fat %, *F* = 11.3, *p* < 0.001, *R*^2^ = 0.617, adjusted *R*^2^ = 0.563			
	Unstandardized		Standardized Coefficients	
	B	Standard Error	*β*	*p*
Experimental group	0.821	0.243	0.486	0.003
SOD (U/L)	0.190	0.093	0.291	0.054
PAB (U/L)	−0.044	0.017	−0.348	0.020
**Liver**				
Predictors	Dependent variable visceral fat %, *F* = 9.6, *p* < 0.001, *R*^2^ = 0.552, adjusted *R*^2^ = 0.523			
	Unstandardized		Standardized Coefficients	
	B	Standard Error	β	*p*
SOD (U/L)	0.002	0.000	0.639	<0.001
O_2_^•−^ (μmol NBT/min/L)	−0.002	0.001	−0.225	0.087
**Adipose tissue**				
Predictors	Dependent variable visceral fat %, *F* = 19.1, *p* < 0.001, *R*^2^ = 0.633, adjusted *R*^2^ = 0.567			
	Unstandardized		Standardized Coefficients	
	B	Standard Error	β	*p*
Experimental group	1.291	0.212	0.818	<0.001
SOD (U/L)	−0.012	0.004	−0.343	0.008
AOPP (μmoL/L)	−0.002	0.001	−0.258	0.036
TOS (μmoL/L)	0.060	0.020	0.422	0.006
TAS (μmoL/L)	−0.002	0.001	−0.327	0.015

MLR—Multiple Linear Regression analysis; SOD—superoxide dismutase; O2^•−^—superoxide anion radical; PAB—pro-oxidant–antioxidant balance; AOPP—advanced oxidation protein products; TOS—total oxidant status; TAS—total antioxidant status.

## Data Availability

The data sets used and/or analyzed during the current study are available from the corresponding author on reasonable request.

## Supplementary Materials

The following supporting information can be downloaded at: https://www.mdpi.com/article/10.3390/antiox12061148/s1. Figure S1. HPLC-MS/MS chromatograms. Table S1: Nutritional information and energetic value of the juices obtained from nutrition labels. Table S2: Correlation between weight and visceral fat percentage in rats. Table S3: Correlation between various parameters and plasma oxidative stress parameter in rats. Table S4: The association between various parameters and oxidative stress parameters in the liver in rats. Table S5: The association between various parameters and oxidative stress parameters in adipose tissue in rats.
