# Supplementary material for: Antioxidative Effects of Black Currant and Cornelian Cherry Juices in Different Tissues of an Experimental Model of Metabolic Syndrome in Rats
Source: Antioxidants (Basel). 2023 May 24;12(6):1148. doi: 10.3390/antiox12061148 (PMC10294917; doi:10.3390/antiox12061148)
Supplement: Supplementary file 1 [file antioxidants-12-01148-s001.zip › antioxidants-2382021-supplementary.pdf]

Table S1. Nutritional information and energetic value of the juices (per 100 mL)*

Nutrients	**BC**	**CC**
Carbohydrates (g)	14	12
Fats (g)	1	0.19
Proteins (g)	0.3	0.48
Energetic value (kcal)	63	52

BC – 100% black currant juice; CC - 100% cornelian cherry juice; *taken from nutrition labels

Table S2. Correlation between weight, visceral fat percentage in rats

		Initial weight	Final weight	Visceral fat (%)
Δ weight	r	-0.347	0.772	0.351
	p-value	0.024	0.000	0.024
Final weight	r			0.428
	p-value			0.005

Table S3. Correlation between various parameter with plasma oxidative stress parameter in rats

		MDA	PAB	AOPP	IMA
Initial weight	r	0.379			
	p-value	0.046			
Final weight	r	0.398			
	p-value	0.036			
Visceral fat (%)	r		0.368		
	p-value		0.038		
SHG	r		-0.338	0.381	0.570
	p-value		0.059	0.035	0.001

MDA - malondialdehyde; PAB - prooxidant antioxidant balance; AOPP – advanced oxidation protein products; IMA - ischemia modified albumin; SHG - sulfhydryl groups;

Table S4. The association between various parameter with oxidative stress parameters in the liver in rats

Parameter		MDA	AOPP	IMA	$O_2^{\cdot-}$	PAB	TOS	TAS	SHG	SOD	PON1
Final weight	r	-0.340									
	p	0.042									
visceral fat (%)	r				-0.402					0.748	0.474
	p				0.015					0.000	0.003
PAB	r		0.423	0.607			0.671	-0.661	0.457		
	p		0.009	0.000			0.000	0.000	0.005		
AOPP	r				-0.378		0.647				
	p				0.021		0.000				
IMA	r						0.346	-0.514	0.461		
	p						0.036	0.001	0.004		
TOS	r		0.647							0.372	
	p		0.000							0.025	
TAS	r						-0.567				
	p						0.000				
TAS/TOS	r		-0.531	-0.423		-0.716	-0.938	0.752		-0.411	
	p		0.001	0.009		0.000	0.000	0.000		0.013	
PON1	r	-0.384	0.482		-0.353					0.565	
	p	0.021	0.003		0.032					0.000	

MDA - malondialdehyde; AOPP – advanced oxidation protein products; IMA - ischemia modified albumin; $O_2^{\cdot-}$ superoxide anion radical; PAB - prooxidant antioxidant balance; TOS - total oxidant status; TAS - total antioxidant status; SHG - sulfhydryl groups; SOD - superoxide dismutase; PON1 – paraoxonase1

Table S5. The association between various parameter with oxidative stress parameters in adipose tissue in rats

Parameter		MDA	AOPP	IMA	$O_2^{\cdot-}$	SH	TOS
Final weight	r			-0.364			
	p			0.032			
Visceral fat (%)	r		-0.338				
	p		0.044				
PAB	r			-0.455			
	p			0.006			
AOPP	r				0.365		
	p				0.026		
TOS	r	0.335		0.346	0.496	0.689	
	p	0.046		0.042	0.002	0.000	
TAS	r	0.381					
	p	0.022					
TAS/TOS	r			-0.459	-0.395	-0.692	-0.925
	p			0.006	0.015	0.000	0.000

MDA - malondialdehyde; AOPP – advanced oxidation protein products; IMA -ischemia modified albumin; O2.- superoxide anion radical; SHG - sulfhydryl groups; PAB -prooxidant antioxidant balance; TOS - total oxidant status; TAS - total antioxidant status;

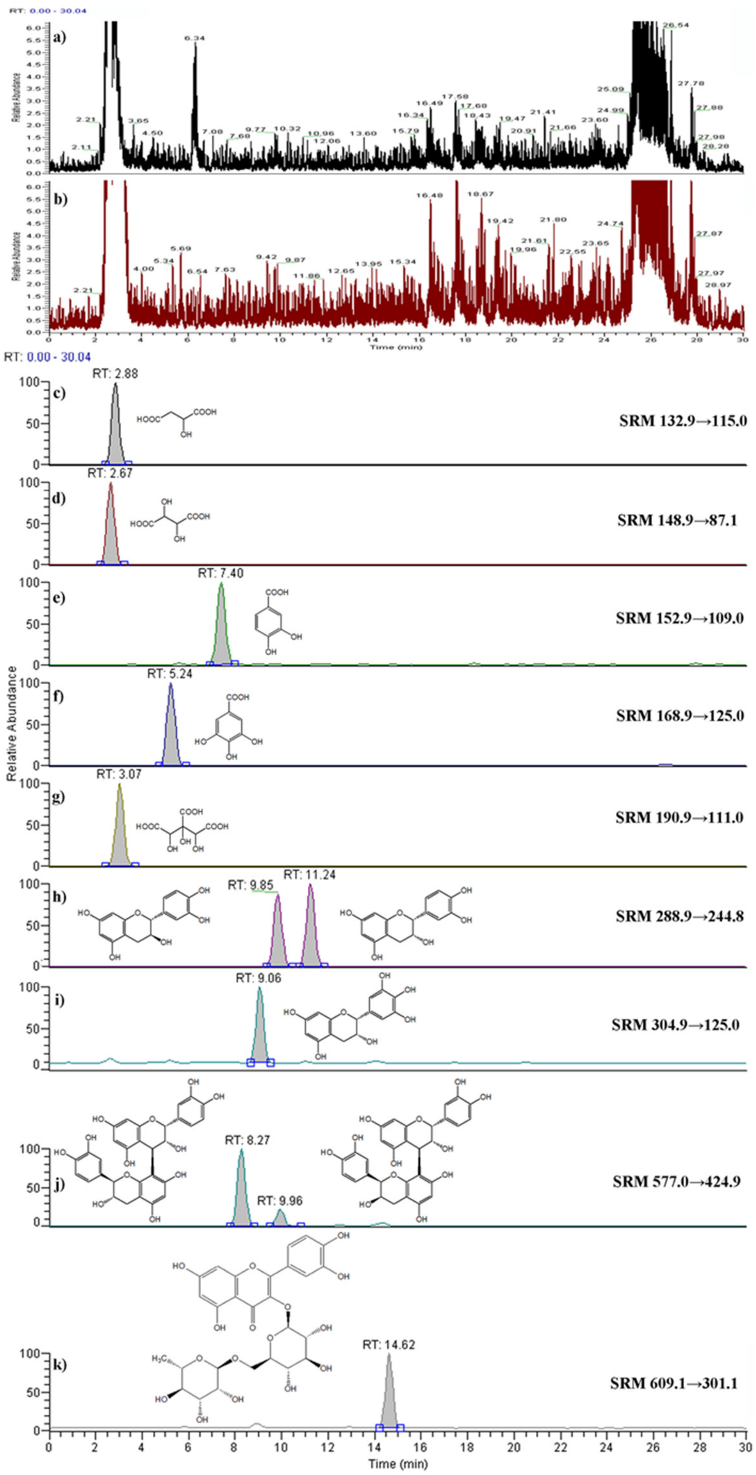

Figure S1. HPLC-MS/MS chromatograms

a) Total ion chromatogram of 100% cornelian cherry juice; b) Total ion chromatogram of 100% black currant juice; c) Malic acid; d) Tartaric acid; e) Protocatechuic acid; f) Gallic acid; g) Citric acid; h) (+)-Catechin and (-)-Epicatechin; i) Epigallocatechin; j) Procyanidin B1 and Procyanidin B2; k) Rutin

RT – retention time; SRM - selective reaction monitoring
